# Minerals, Essential Oils, and Biological Properties of *Melissa officinalis* L.

**DOI:** 10.3390/plants10061066

**Published:** 2021-05-26

**Authors:** Fahima Abdellatif, Muhammad Akram, Samir Begaa, Mohammed Messaoudi, Adel Benarfa, Chukwuebuka Egbuna, Hamza Ouakouak, Aicha Hassani, Barbara Sawicka, Walaa Fikry Mohammed Elbossaty, Jesus Simal-Gandara

**Affiliations:** 1 Research Laboratory on Bioactive Products and Biomass Valorization, Higher Normal School, Kouba P.O. Box 92, Algiers 16308, Algeria; zina_fahima@yahoo.fr (F.A.); aicha_hassani2@yahoo.fr (A.H.); 2 Department of Eastern Medicine, Directorate of Medical Sciences, Government College University Faisalabad, Faisalabad 38000, Pakistan; makram_0451@hotmail.com; 3 Nuclear Research Centre of Birine, Ain Oussera P.O. Box 180, Djelfa 17200, Algeria; samirbegaa@yahoo.fr; 4 Chemistry Department, University of Hamma Lakhdar, B.P.789, El-Oued 39000, Algeria; hamza39ouakouak@gmail.com; 5 Technical Platform of Physico-Chemical Analysis (PTAPC-Laghouat-CRAPC), University of Amar Telidji, Road of El kheneg, Laghouat 03000, Algeria; adel.benarfa@gmail.com; 6 Nutritional Biochemistry and Toxicology Unit, World Bank Africa Centre of Excellence, Centre for Public Health and Toxicological Research (ACE-PUTOR), Department of Biochemistry, University of Port-Harcourt, Rivers State 500001, Nigeria; egbunachukwuebuka@gmail.com; 7 Department of Plant Production Technology and Commodities Science, University of Life Science in Lublin, Akademicka 15 Str., 20-950 Lublin, Poland; barbara.sawicka@gmail.com; 8 Department of Chemistry, Faculty of Science, Damietta University, Damietta 34517, Egypt; walaafikry1985@gmail.com; 9 Nutrition and Bromatology Group, Department of Analytical Chemistry and Food Science, Faculty of Food Science and Technology, University of Vigo—Ourense Campus, 32004 Ourense, Spain

**Keywords:** *Melissa officinalis* L., medicinal plants, mineral content, essential oils composition, antibacterial activity, antioxidant activity

## Abstract

This study describes the minerals elements, chemical composition, antioxidant and antimicrobial activities of Algerian *Melissa officinalis* plant. The essential oil (EO) was extracted by hydrodistillation (HD) using a Clevenger-type apparatus of dry leaves of *M. officinalis* and was analyzed by two techniques, gas chromatography coupled with flame ionization (GC-FID) and gas chromatography coupled with mass spectrometry (GC-MS). Eighteen minerals comprising both macro- and microelements (As, Br, K, La, Na, Sb, Sm, Ba, Ca, Ce, Co, Cr, Cs, Fe, Rb, Sc, Th, and Zn) were determined using neutron activation analysis technique for the first time from Algerian *Melissa officinalis* plant. Seventy-eight compounds were identified in the essential oil, representing 94.090% of the total oil and the yields were 0.470%. The major component was geranial (45.060%). Other predominant components were neral (31.720%) and citronellal (6.420%). The essential oil presented high antimicrobial activity against microorganisms, mainly five human pathogenic bacteria, one yeast, *Candida albicans*, and two phytopathogenic fungi. The results can be used as a source of information for the pharmaceutical industry and medical research.

## 1. Introduction

**Figure 1:**
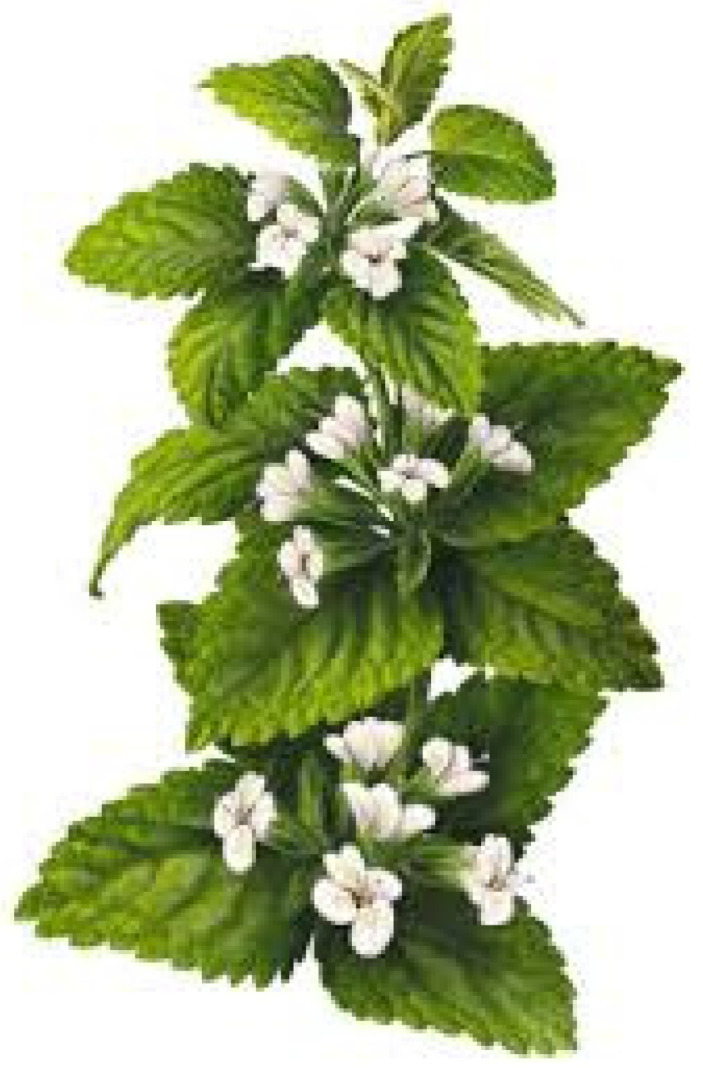
The aerial parts of *M. officinalis*.

*Melissa officinalis* L., commonly known as lemon balm, honey balm, balm mint, garden balm, or common balm is a perennial herbaceous plant that belongs to the family Lamiaceae (mint family). It is found predominantly in the Mediterranean region of the world and elsewhere such as Central Asia, Iran, Europe, Serbia, America, and Africa [1,2,3]. *M. officinalis* has the ability to grow fast and establish itself in its natural habitat such that some gardeners call it a weed [3]. The average height of the matured plant extends between 70–150 cm [4]. The leaves are dark greenish in color and ovate (Figure 1) with a slight lemon scent that resembles the smell of mint. In the summer, its small white flowers appear and full of nectar that attracts bees [5]. *M. officinalis* leaves are edible (as a vegetable) and have been used as food and medicine by man for many centuries [6,7]. The edible, scented and functional properties of *M. officinalis* make it a plant of choice for application in food and pharmaceutical industries. *M. officinalis* extract is medicinal and most countries use it in their traditional systems of medicine to treat many diseases [8,9,10,11,12,13].

In Algeria, *M. officinalis* is used as integrated pharmaceutical treatment option for the treatment of headache, indigestion, abdominal cramps, heart failure, diabetes and bacterial viral infections [1,14]. Scientific research has confirmed that the medicinal benefits of *M. officinalis* is due to the presence of wide range of secondary metabolites such as flavonoids, phenolic acid, and terpenes [15]. Other secondary metabolites of *M. officinalis* are mostly from its essential oil (EO), which includes eugenol, octinol, octin, octinone, citral, hexenol, and haramin. In addition, containing a large amount of rosmarinic acid (36.5 per g of the plant), it is used in the treatment of many diseases [2].

*M. officinalis* essential oil has been reported to be medicinal [16,17]. Essential oils are concentrated hydrophobic oils that contain some volatile chemical compounds which are sensitive to light, temperature, oxygen and moisture [18]. It can be extracted from plants by distillation, solvent extraction, oil absolute extraction and the use of resin binder or by cold pressing [19,20,21]. EO is used in industry where it is added to food, and in the perfume or cosmetics industry for its scents or fragrances [22,23,24,25]. The medicinal features of EOs are varied. Based on this, EOs are best classified or grouped based on their plant sources. For instance, it is more useful and distinguishing to say EO of chamomile, EO of peppermint, EO of tea tree, EO of lavender or EO of *M. officinalis*. EOs are not all the same, the biochemical properties of an EO are a direct reflection of the plant secondary metabolite composition. Again, the commercially available EOs even from the same plant sources may differ in composition. This is because the end product of an EO is affected by the choice of extraction method and solvents, the carrier oil used, the method and duration of storage, and environmental factors [26,27]. Adding EOs to a carrier oil such as vegetable oil, olive oil, coconut oil, castor oil, rosemary oil or other kinds of oil entails that the EO will no longer be pure and that its effects will be a sum of the total secondary metabolites in both the EO and the carrier oil.

The concept of aromatherapy is as old as human civilization. Aromatherapy or EO therapy involves the use of EOs from plant extracts for healing purposes. EOs have been reported to be effective against high blood pressure, stress, depression, management of pain and so on [28,29]. Although, despite the medicinal values of EO, it is advisable to apply caution in their use because some may cause skin irritation and some kinds of allergy [25], probably because of the presence of toxic metabolite or poison. This is why it is helpful to characterize EO (usually by gas chromatography methods) from different plants so as to unravel its phytochemical composition.

In the present study, we aimed to determine the chemical composition of the essential oil of wild-growing *Melissa officinalis* plant as well as the antioxidant activity of EOs, antimicrobial activities and mineral elements for the first time for this plant.

## 2. Results

### 2.1. Mineral Elemental Analysis

To our knowledge and until now, there are no scientific researches issued focusing on the multielement (mineral compounds) composition of *M. officinalis* despite the importance of trace elements in various human metabolic processes and their contribution of significantly to human health [30], the present work attempts to fill that gap using the high detectability instrumental neutron activation analysis technique (INAA). The mineral content of an Algiers *M. officinalis* sample was determined using the INAA technique. It was found to contain 18 elements, which include both macro- and microelements (As, Br, K, La, Na, Sb, Sm, Ba, Ca, Ce, Co, Cr, Cs, Fe, Rb, Sc, Th, and Zn).

In order to ensure that quality of analytical data and chemical element contents were attained, we used two standards (GSV-4 GBW 07605 and NIST-SRM 1573a) [31]. The mineral chemical elements’ contents of the standards were measured in order to evaluate the technique performance. Table 1 and Table 2 represent a comparison between determined values and certified values of these standards. In most cases herein, the determined values on this step were within 10% range of those of the certified values. Furthermore, this evaluation showed significant quality of results as revealed by the statistical evaluation, where U-score values were all accepted.

In order to determine the variability of the content of minerals in the plant depending on the change in concentration, six different samples of *M. officinalis* were collected and the minerals were measured (Table 3).

Precision means the closeness of agreement between single analytical results (scattering of results) when a given procedure is applied to multiple, independent, homogeneous determinations on a homogeneous sample. The most common measures of precision are standard deviation (S), relative standard deviation (RSD) or coefficient of variation (V). Precision includes two concepts: repeatability and reproducibility. Acceptance criteria: The CV of the results of the ingredient content should not be greater than 3%. The CV of the results at the trace level should not be greater than 15%. The difference between two independent results must not exceed the values given in the accuracy tables. Both measurements are valid with a 95% probability. This means that on average when 20 determinations are performed, only one outlier can be acceded. Interlaboratory variability, defined as the coefficient of variation R or RSD%, is the most frequently used qualitative parameter to compare the accuracy of analytical methods. In general, typically the RSD% decreases exponentially with increasing concentration of the measured variable. In methods of analyzing the main ingredients of food, which are in the range from 0.01 g·100 g^−1^ to 10 g·100 g^−1^, the coefficient of variation ranges from 0.1–10%. The greater the RSD% for the value, the poorer the repeatability of the method. To compare the performance of the methods in turn, the extreme RSD% values for each method in the team studies are summarized (Table 3).

Reproducibility allows you to assess whether a given method leads to the same results in different laboratories with different analysts on different equipment and under different conditions, of course with the parameters in the description of the method. The tests are carried out in the same way as in the case of repeatability. The mean value, confidence interval, standard deviation, relative standard deviation and coefficient of variation of the obtained results are determined.

In terms of stability, the chemical elements contained in the *M. officinalis* leaves can be arranged as follows: K > Na > La > Ca > Zn > Fe > Sm > Br > Rb > As > Cs > Sb > Sc > Ba > Co > Ce > Th > Cr. The most variable element in the *M.officinalis* turned out to be chromium and the most stable was potassium (Table 3).

In this study, the results of mineral content of *M. officinalis* showed that it is a rich source of mineral compounds such as Ca (28,385 mg/kg), K (18,474.000 mg kg^−1^), Fe (1491.000 mg kg^−1^), Na (897.000 mg kg^−1^) and Zn (51.400 mg kg^−1^). Hence, it might be concluded and confirmed from this study that this plant would not only serve as a flavoring agent but also a good source of several essential mineral elements.

### 2.2. Chemical Composition of Melissa officinalis EO

The oil of the leaves of *M. officinalis* isolated by hydrodistillation was of pale yellow color with a lemony smell, with total yield of 0.470% *w*/*w* on dry weight basis.

Qualitative and quantitative analytical results were obtained using both GC and GC-MS techniques. Table 4 shows the compounds identified in the oil of *M. officinalis* in order of elution on HP5 capillary column, the percentage content of the individual components, retention indices and chemical class distribution are summarized.

Seventy eight compounds were identified, accounting for 94.090% of the total oil. This oil was characterized by very high percentage of oxygenated monoterpenes (84.980%) in which, neral (31.720%), geranial (45.060%) and citronellal (6.420%) were the major components.

In contrast, the sesquiterpene hydrocarbons fraction was lower (6.180%) represented by β-curcumene (1.590%), α-copaene (3.21%) and β-caryophyllene (2.20%) were detected in higher concentration than the oxygenated sesquiterpenes, such as caryophyllene oxide (0.310%).

The above results show that our oil was characterized by the presence of three dominating components in monoterpenoid family type aldehyds, and an important fraction includes neral (31.720%), geranial (45.060%) and citronellal (6.42%).

### 2.3. Antioxidant Character of M. officinalis

The antioxidant activity of the EO of *M. officinalis* was evaluated by the DPPH method. IC_50_ is inversely related to the antioxidant capacity of a compound or essential oil, as it expresses the amount of antioxidant needed to decrease the concentration of the free radical by 50%. The lower the IC_50_ value, the greater the antioxidant activity. The IC_50_ values for the EO as well as for the reference compounds, BHT and BHA, alpha-tocopherol and vitamin E were presented in the Table 5. The IC_50_ of *M. officinalis* essential oil was found to be more than 44,000 µg/mL.

**Figure 2:**
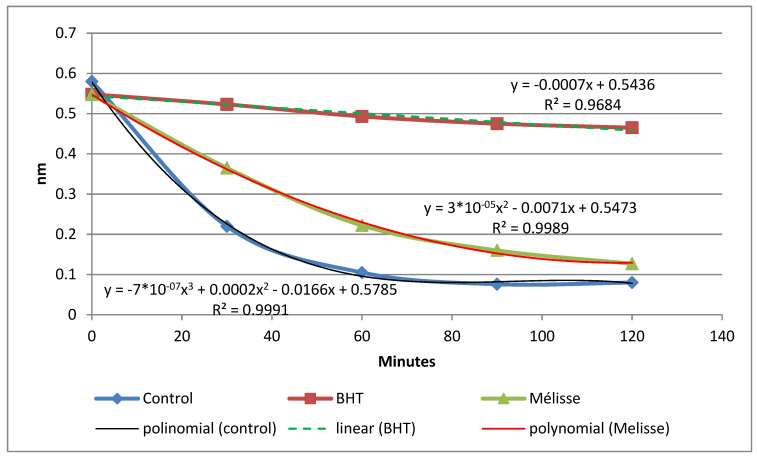
Bleaching kinetics of β-carotene at 470 nm in the absence and presence of the essential oil of *M. officinalis* and standard antioxidant (BHT).

In this test β-carotene/linoleic acid method, the ability of the EO of *M. officinalis* and the standard (BHT) to slow the rate of lipid oxidation were evaluated by measuring the decrease in absorbance over time (Figure 2). BHT and EO are able to inhibit β-carotene bleaching by scavenging free radicals derived from linoleic acid. After 120 min, the absorbance of the control at 470 nm decreased, but this decrease was less rapid than in the OE tested.

The decrease in lipid oxidation in this standard antioxidant (BHT) was according to the linear polynomial regression model (y = −0.0007x + 0.5436a), with R^2^ = 0.9684.

The regression model for EO of *M. officinalis*, was determined according to the second degree curvilinear parabolic equation the second degree curvilinear parabolic equation (y = 3 × 10^−5^ x^2^ − 0.0071x + 0.5473a), with the coefficient of determination R^2^ = 0.9989. This means that the regression equation is highly reliable (99.89% confidence) and you can trust the calculation.

In the control object, this decrease occurred according to the three-degree equation (y = −7 × 10^−7^ x^3^ + 0.0002x^2^ − 0.0166x + 0.5785a) with R^2^ = 0.9991 (Figure 2).

The coefficient of determination denoted as R^2^ indicates the accuracy of modeling, the actual data points by the regression equation. The value of the R^2^ coefficient is a number between 0 and 1, where values closer to 1 indicate greater accuracy of the model. The value of the R^2^ coefficient equal 1, which notes a perfect model, very unlikely in real situations, considering the complexity of interdependencies between various factors and unknown variables. Therefore, regression models were created with the highest possible value of the R^2^ coefficient, with a value close to 1.

Regression analysis creates a mathematical function that describes the relationship between the predictor (s) and the dependent variable. The coefficient of determination informs us what part of the variability (variance) of the explained variable in the sample coincides with the correlations with the variables included in the model. It is therefore a measure of the extent to which the model fits into the sample. The coefficient of determination assumes values in the range (0; 1) if there is an intercept in the model, and the method of least squares is used to estimate the parameters. Its values are most often expressed as a percentage.

The model fit is interpreted as better, where the R² value is closer to one (1.0). Thus, our regression models turned out to be very closely aligned with the actual values.

That research showed, that EO has a weak antioxidant activity. It was shown on the basis of high IC_50_ value (>44,000 µg/mL), compared to Vitamin C (7.250 ± 0.030 µg/mL) and E (9.560 ± 0.070 µg/mL). EO of *M. officinalis* inhibits in a less efficient way compared to the oxidation of linoleic acid by BHT. Therefore, the EO of *M. officinalis* tested has less inhibition (AA = 15.860 ± 0.700%), compared to the oxidation of the β-carotene/linoleic acid as system of the reference substances (BHT with AA activity of 83.560 ± 2.130%) (Table 5).

### 2.4. Antimicrobial Characterization

Antimicrobial activity was evaluated with respect to EO of *M. officinalis* leaves by the paper disc diffusion method. The results showed that *M. officinalis* EO has antibacterial activities (Table 6). The EO showed strong activity against all strains tested with very low zones of inhibition (10 mm) for Gram-positive bacteria *Bacillus subtilis* from *M. officinalis* EO. For pathogenic Gram-negative bacteria, zones of inhibition were observed, which ranged between 10–50 mm. In general, the EOs were more active against Gram-negative bacteria than Gram-positive bacteria (Table 6).

The EO of *M. officinalis* also showed good antiyeast activity against *Candida albicans* that is pathogenic to humans. The phytopathogenic fungi tested, *Fusarium oxysporum albedinis* and *Fusarium oxyspourum lini* are the agents of *Fusarium wilt* of date palm and flax, respectively. The research of the antifungal activity showed that the EOs from *M. officinalis* are more potent (20–50 mm) than Nystatin (20–24 mm), which our reference antifungal used (Table 6).

## 3. Discussion

In human metabolism, it is well known that the human body needs certain essential minerals for its growth; in general, they are indispensable for a healthy human nutrition, with important physiological roles [32].The result of mineral content analysis of *M. officinalis* leaves presented in Table 1 shows that *M. officinalis* leaf contains macro and microminerals, and that the most common element was K (16,412), followed by iron (282), Rb (75), Ba (58), Na (51), Zn (28). Shekarchi et al. stated that potassium was more common (17.275 ppm), followed by Ca (5698 ppm), Mg (5550 ppm), Fe (119.4 ppm), Na (83.34 ppm), and Zn (29.163 ppm). Lower values were recorded for Mn 16.41 ppm, Cu 6.559 and Ni 1.067 [33,34]. The difference in the concentration of these minerals among various studies is due to the plant’s ability to absorb nutrients from the soil, the soil’s mineral content, sample preparation, irradiation and the counting system [35].By comparison between *M. officinalis* leaves and the tomato leaves, it was found that there were some elements in abundance while some were absent. Tomato leaves contain Br, Fe, Na, and Zn, while *M. officinalis* leaves contains K, Rb, Co, La, and Sm. On the other hand, compared with tea leaves, we found they contain Br, Co, K, and La, while *M. officinalis* leaves contain Ba, Fe, Na, Rb, Sc, and Zn. When z-score was measured, it was found to be less than 2, which means that the estimation of these elements in all plants used was correct. Hence, it might be concluded and confirmed from this study, that this plant is not only a flavoring agent but also a good source of several essential nutrient elements.

The result obtained for the chemical composition analysis of *M. officinalis* EO from Algiers (Table 4) revealed that they contain varying quantities of different phytochemicals. Notable phytochemicals found include geranial (45.060%), neral (31.720%), citronellal (6.420%), curcumene-(β) (1.590%), and (β)-caryophyllene (2.200%). The result is analogous to the results that were obtained in other countries such as Serbia [17], Slovakia [36], Egypt [37], and France [38]. Again, varying quantities of the phytochemicals were found in EOs from other regions considered in this study (Table 4). From these studies, it is clear that the content of essential oils in *M. officinalis* varies from one place to another according to the geographical diversity as they are similar in countries that have an almost similar geographical area, and the proportions differed in countries that are not geographically similar, among other factors [7,39,40].

The low antioxidant properties of EO of *M. officinalis* (Table 5) could be attributed to the low contents of volatile phenolic compounds such as camphor and carvacrol, which in part explains its low antioxidant activity in two test methods (DPPH and β-carotene).

Finally, the results of antimicrobial studies of EO against different strains of both Gram-positive and Gram-negative bacteria and fungi proved that it has antimicrobial activities. This result is in agreement with the report of Mimica-Dukic [17] who stated that EO extracted from *M. officinalis* have antibacterial and antifungal characterization in a dose-dependent manner.

## 4. Materials and Methods

### 4.1. Plant Material and Sample Preparation

The sample of *M. officinalis* leaves (Figure 1) was collected in May 2018 at Algiers in north Algeria (36°46′ N, 3°03′ E). The species was identified at the Laboratory of Bioactive Products and Biomass Valorization Research, ENS Kouba, and was confirmed in the botanical department of National Institute Agronomic of Algiers (NIA), Algeria.

### 4.2. Detection of Elements in M. officinalis

The studied samples were prepared as described by Benarfa et al. [30] with slight modification. The samples were washed well with tap water and then with deionized water in order to remove soil particles and dust, then dried carefully for 48 h in an oven at 30°C. Next, the dried samples were ground manually using an agate mortar and pestle, before sieving them through a stainless steel sieve (150 µm mesh size) and then placing in an aluminum irradiation capsules as well as the standards. In this study, two standard reference materials were used. The first one was NIST-SRM 1573a (tomato leaves) from the National Institute of Standard and Technology (NIST), and the second was Chinese tea leaves (GBW 07605, National Research Center for CRM, Lang fang, China). The capsule prepared was subjected to thermal neutron flux for two hours. The work was carried out by ray gamma detection using high-purity germanium (HPGe) cooled using liquid nitrogen. The system has the following characteristics: relative efficiency: 35%, FWHM 1.8 keV for the 1332.5 keV γ-peak of ^60^Co. The first measurement was 2 h after 3 day; the second measurement for the long-lived radionuclides was after day 18 for a collection time of 4 h.

In this experiment, NIST-SRM 1573-certified reference materials were used to determine chemical concentrations of elements using relative techniques, and GBW 07605 for quality control.

### 4.3. Identification of Essential Oils in Melissa officinalis

#### 4.3.1. Isolation of Essential Oils

The essential oils were extracted from the leaves of the plant using the Clevenger-type apparatus according to the European Pharmacopeia [41] was employed. A quantity of 100 g of *M. officinalis* for 2 L of distilled water was used to perform the hydrodistillation for 180 min. The essential oils were dried using anhydrous sodium persulfate and stored in the dark at 4 °C until use. The weight of the volatile oils was calculated by weight according to the following Equation (1):

(1) $$\mathrm{Essential}\;\mathrm{oil}\;\left(\%\right)=\frac{\mathrm{weight}\;\mathrm{of}\;\mathrm{the}\;\mathrm{volatile}\;\mathrm{essential}\;\mathrm{oil}\;\mathrm{extracted}\;}{\mathrm{weight}\;\mathrm{of}\;\mathrm{sample}\;\mathrm{taken}\;}\times 100$$

#### 4.3.2. GC-FID Analysis

A Hewlett Packard HP5890 series II GC-FID system was used for chromatography analysis, fitted with a fused silica capillary column with apolar stationary phase HP5MS (30 m × 0.25 mm, 0.25 μm film thickness) and polar stationary phase HPWax (30 m × 0.15 mm, 0.25 μm film thickness). The temperature program was 60 °C for 5 min increased at 3 °C/min to 250 °C for 5 min. Injection was performed at 250 °C in the split mode; 1/50. 0.1 μL of the oil was injected. A flow rate of 1 mL/min carrier gas (N_2_) was used.

The percentage composition of the individual components was recorded from electronic integration measurements using flam ionization detection (FID; 260 °C). In order to determine retentions indices (RI), a series of n-alkanes (C_5_–C_28_) mixtures were analyzed under the same operative conditions on HP5MS column; the retention indices were calculated following Van den Dool methods [42] Equation (2).

(2) $$RI=100n+100\times \frac{\left(tr_{comp}-tr_{C_{n}}\right)}{\left(tr_{C_{n+1}}-tr_{C_{n}}\right)}$$

$tr_{comp}$: retention time of compound; $tr_{C_{n}}$: retention time of $C_{n}$ alkane,$tr_{\left(C_{n+1}\right)}$: rention time of $C_{n+1}$ alkane

#### 4.3.3. GC/MS Analysis

The volatile compounds were analyzed by gas chromatography coupled to mass spectrometry (GC-MS). Analysis was performed on a GC/MS Hewlett Packard HP5890 series II chromatograph coupled to a HP MSD5971 mass spectrometer using fused-silica-capillary column. Thenon-polar column was DB5 (30 m × 0.25 mm × 0.25 µm film thickness). GC-MS spectra were obtained using the following conditions: He (Helium) as carrier gas at flow rate of 1 mL/min; split mode 1:50; 0.1µL as injected volume; 250 °C as injection temperature. The oven temperature program was 60 °C for 5 min increasing at 3 °C/min towards 250 °C and held at 250 °C for 10 min. The ionization mode used was electronic impact at 70 eV. The identification was confirmed by comparison of the mass spectral with those stored in the MS database (National Institute of Standards and Technology NIST08 and Wiley libraries) and also by comparison with mass spectra from literature data [43].

### 4.4. Determination of Antioxidant Character for Melissa officinalis

#### 4.4.1. Evaluation of the Free Radical Scavenging Activity by the DPPH Method

This is the most widely used method to assess the antioxidant activity of herbal drugs. This test aims to measure the ability of the oil to scavenge the relatively stable radical; 1,1-diphenyl-1-picrylhydrazyl (DPPH) [44]. The scavenging of the free radicals of DPPH causes a color change of the initial solution from dark purple to yellow following the reduction of DPPH to DPPH-H (diphenyl-picrylhydrazine). Five milliliters of a freshly prepared ethanolic solution of DPPH (0.004%), 50 µL of various concentrations of each EO (2000–44,000 µg/mL) and of each standard (5–16,000 µg/mL) were added. The mixtures are vortexed and incubated in the dark at room temperature for 30 min. The disappearance of DPPH was followed spectrophotometrically at 517 nm against a blank (ethanol solution) using a spectrophotometer (JASCO-V53).

The IC50 inhibitory concentration value represents the dose of EO that causes the neutralization of 50% of the DPPH radicals. The IC50, used as an estimate of the antioxidant activity by DPPH, is estimated by extrapolation by plotting the percentage inhibition (I%) curve as a function of the concentrations. All tests were performed in three runs and IC50 values were reported as the mean ± SD.

#### 4.4.2. Evaluation of the Antioxidant Activity of EO by the β-Carotene/Linoleic Acid Method

The β-carotene/linoleic acid method is one of the complementary methods used for screening of antioxidant substances. It is based on the principle that the unsaturated fatty acid, linoleic acid, spontaneously oxidizes with the reactive oxygen species (ROS) present in oxygen-rich water. Then the reaction products trigger the transformation of β-carotene into its colorless form. The degree of discoloration is measured spectrophotometrically and used as an estimate for antioxidant activity (AA) [45,46]. The stock solution of β-carotene/linoleic acid emulsion mixture was prepared as follows: 1.0 mg of β-carotene crystals was dissolved in 10 mL of chloroform and 1 mL of this solution was transferred to a flask containing 20 mg of linoleic acid and 200 mg of Tween-40. After complete removal of chloroform by evaporation (using a rotary evaporator at 40 °C), 50 mL of distilled hydrogen peroxide was added with vigorous stirring to form an emulsion. Five milliliters of the emulsion was added to 0.2 mL of the antioxidant solution in test tubes, containing 350 µL of EO diluted in ethanol. The final concentration was 2 g/L. The solution was homogenized, and absorbance measurements were taken at 470 nm immediately after the addition of the emulsion to the antioxidant solution. After vigorous shaking, the tubes were incubated at 50 °C for 2 h with shaking, absorbance measurements were taken at 15 min intervals until the absorbance of the read control got below 0.03. All determinations were made with three repetitions.

A control consisting of 20 mg of linoleic acid, 200 mg of Tween 40 and 50 mL of hydrogen peroxide was used to calibrate the spectrophotometer. A negative control tube was prepared by replacing the EO with ethanol. The absorbance was finally measured at 470 nm against a blank (emulsion without β-carotene). Antioxidant activities (*AA*%) were calculated using the following Equation (3):

(3) $$AA\%=\left(1-\frac{A_{0}-A_{t}}{{A^{\prime }}_{0}-{A^{\prime }}_{t}}\right)\times 100\;$$

where: *A*_0_, *A*_0_’—respective absorbance of the sample and of the control at t = 0 min; *A*_t_, *A*_t_’—respective absorbance of the sample and of the control at t = 2 h.

#### 4.4.3. Antimicrobial Activity

(a) Tested Strains

For the bacteria four resistant Gram-negative strains, as: *Klebsiella pneumoniae* (CIP 8291), *Escherichia coli* (ATCC 10536), *Salmonella entirica* (CIP 813) and *Pseudomonas aeruginosa* (CIP A22), and two Gram-positive strains *Staphylococcus aureus* (CIP 7625) and *Bacillus subtilis* (ATCC 6633); for mold strains: *Mucor ramannianus* (NRRL 6606), *Fusarium oxysporum albedinis* (CURZA)and *Fusarium oxysporum lini* (CINRA) and yeasts *Candida albicans* (IPA200) and *Saccharomyces cerevisiae* (ATCC 4226). All tested strains were provided by the microbiology laboratory of the higher normal school of Kouba, Algeria, where these experiments were done. These bacteria except *Bacillus subtilis* were chosen because they are the most common Gram-positive and Gram-negative bacteria found in nosocomial infection. We also used antibiotic as control: levofloxacin for bacteria, nystatin for fungus.

(b) Disk Diffusion Method

The paper-disk diffusion method [47] was employed for the determination of antimicrobial activity of the essential oil. Microbial suspensions were prepared in sterile 0.9% saline and adjusted as inoculum to a final concentration of 1.0 × 108 CFU/mL. A volume of 20 mL of Mueller–Hinton agar and Sabouraud, respectively, for bacterial and fungal strains was inoculated with 20 μL of microbial suspension and then poured into a Petri dish. The plates were left at room temperature for 30 min to allow the culture media to solidify. Each paper disk of 6 mm diameter was impregnated with 35 μg of essential oil solution (in methanol) and then applied manually on the surface of the agar plates inoculated with microorganisms.

Ampicillin and nalidixic acid (30 μg/disk) were used as positive reference standards to determine the sensitivity of Gram-positive and Gram-negative bacteria species, respectively. Nystatin (30 μg/disk) was used as positive reference standard to determine the sensitivity of fungi and yeasts species. The plates were kept at 4 °C for 2 h to allow diffusion, and then incubated for 24 h at 37 °C for bacteria, and 48 h at 30 °C for yeasts and fungi. The antimicrobial activity was determined by measuring with a caliper the diameters of inhibition zones, including disk diameter (6 mm). All tests were carried out in triplicate [1].

### 4.5. Statistical Analysis

Statistical development of results was performed by analysis of variance (ANOVA), analysis of regression, and correlation analysis. The significance of the sources of variation was tested with test “F” Fischer-Snedecor, and the importance of differences between compared averages was made using Tukey’s confidence intervals [48]. Analysis of variance, regression analysis, and correlation calculation were performed in SAS^®^ statistical package [49].

The study used among others mean, range: minimum and maximum, standard deviation, relative statistical deviation (RSD) and coefficients of variability (CV). The results of examining some features were subjected to regression analysis, i.e., regression models were created. The idea of regression is to forecast data for a certain variable based on other variables. With the help of regression analysis, regression models were constructed, which will predict the value or level of a given feature with the assumed statistical error. Using the least squares method, regression lines and trend lines for the collected data were determined and used to estimate both linear and nonlinear relationships. Function parameters were determined using the least-squares method, and the significance was verified with Student’s *t*-test [48].

## 5. Conclusions

The leaves *of M. officinalis* are rich in both micro- and macromineral compounds such as K, Ca, Fe, among many others. The essential oil of this species contains varying concentrations of phytochemicals and was characterized by its high content of monoterpenoids with citral being the predominant one (76.780%). The EO of *M. officinalis* has antibacterial and antifungal activities, which suggests that it may be of therapeutic importance. In contrast, *M. officinalis* essential oils have lower antioxidant properties than expected. The results of the current work will be beneficial and can be used as a reference by researchers and specialists to enrich the medicinal herbs database.

## Figures and Tables

**Table 1:** Comparison of measured values with certified values in standard reference material of GBW 07605 (mg/kg), values represent means ± standard error (n = 3).

Elements	GBW 07605			
	Our Work Measured Value	Certified Value	Z-Score	U-Score
Ba	58.000 ± 14.000	58.000 ± 3.000	0.000	0.000
Br	3.091.000 ± 0.078	3.400 ± 0.400	0.770	0.760
Co	0.170 ± 0.030	0.180 ± 0.020	0.500	0.280
Fe	282.000 ± 33.000	264.000 ± 10.000	1.800	0.520
K	16,412.000 ± 339.000	16,600.000 ± 600.000	0.310	0.270
La	0.590 ± 0.020	0.600 ± 0.030	0.330	0.280
Na	51.200 ± 10.000	44.000 ± 4.000	1.800	0.670
Rb	75.000 ± 7.000	74.000 ± 4.000	0.250	0.120
Sc	0.087 ± 0.014	0.085 ± 0.009	0.220	0.120
Sm	0.077 ± 0.013	0.085 ± 0.017	0.470	0.370
Zn	28.000 ± 2.000	26.003 ± 0.900	1.890	0.780

**Table 2:** Comparison of measured values with certified values in standard reference material of NIST1573a (mg/kg). Values represent means ± standard error (n = 3).

Elements	NIST-SRM 1573a			
	Our Work Measured Value	Certified Value	Z-Score	U-Score
Ba	63.800 ± 13.000	(63.000)	-	0.060
Br	1177.000 ± 142.000	(1300.000)	-	0.870
Co	0.590 ± 0.120	0.570 ± 0.020	1.000	0.160
Fe	355.000 ± 40.000	368.000 ± 7.000	1.860	0.320
K	27,309.000 ± 1018.000	27,000.000 ± 500.000	0.060	0.060
La	2.33.000 ± 0.14.000	(2.300)	-	0.210
Na	133.700 ± 10.500	136.000 ± 4.000	0.580	0.200
Rb	15.000 ± 2.000	14.890 ± 0.270	0.410	0.050
Sc	0.097 ± 0.010	(0.100)	-	0.300
Sm	0.209 ± 0.044	(0.190)	-	0.430
Zn	29.600 ± 2.000	30.900 ± 0.700	1.860	0.610

**Table 3:** Elemental concentration in *M. officinalis* (mg/kg) (n = 6). All values are expressed in dry weight.

Elements	Means	Maximum	Minimum	Standard Deviations	CV (%)
As	0.700	0.799	0.601	0.099	14.143
Br	12.700	14.300	11.100	1.600	12.598
K	18,474.000	19,002	17,946.000	528.000	2.858
La	1.528	1.619	1.437	0.091	5.955
Na	897.000	949.000	845.000	52.000	5.797
Sb	0.098	0.112	0.084	0.014	14.286
Sm	0.241	0.267	0.215	0.026	10.705
Ba	78.000	92.000	64.000	14.000	17.949
Ca	28,385.000	30,359.000	26,411.000	1974.000	6.954
Ce	3.320	4.130	2.510	0.810	24.398
Co	0.515	0.615	0.416	0.1000	19.320
Cr	12.300	14.000	10.600	1.700	138.211
Cs	0.277	0.319	0.235	0.042	15.162
Fe	1491.000	1635.000	1347.000	144.000	9.658
Rb	9.200	10.400	8.000	1.200	13.043
Sc	0.267	0.303	0.231	0.036	14.483
Th	0.326	0.406	0.246	0.080	24.540
Zn	51.400	55.600	47.200	4.200	8.171

**Table 4:** Chemical composition of the essential oil of the Algerian *M. officinalis* leaves.

N^o^	Compounds	RI_réf_	RI_n__p_	RI_p_	%
1	1-Octen-3-one		972	-	0.060
2	1-Octen-3-ol		978	-	0.140
3	6-Methyl-5-hepten-2-one	981	982	1375	0.100
4	α-Terpinene	1014	1016	1245	0.060
5	(Z)-β-Ocimene	1032	1030	1229	0.150
6	(E)-β-Ocimene	1044	1042	1250	0.280
7	*Cis*-linalool oxide	1068	1068	1423	0.090
8	Linalool	1095	1096	1508	0.100
9	Nonanal	1100	1100	1392	1.120
10	*Cis*-rose oxide	1106	1103	1352	0.060
11	*Trans*-rose oxide	1122	1120	1370	0.070
12	Isopulegol(neo)	1144	1137	1573	0.430
13	Citronellal	1148	1147	1465	6.420
14	Nerol-oxide	1154	1154	-	0.100
15	(Z)-Isocitral	1160	1164	-	0.070
16	Terpinene-4-ol	1174	1170	1628	tr
17	Verbanol neo	1182	1182	-	tr
18	Dihydrocarveol	1192	1192	1713	tr
19	Verbenone	1204	1204	1733	tr
20	Neral	1235	1235	1680	31.720
21	Geraniol	1249	1249	1837	0.120
22	Geranial	1264	1266	1732	45.060
23	Methyl nerolate	1280	1281	-	0.250
24	Methyl geranate	1322	1324	-	tr
25	Dihydrocarveol acetate (iso)	1326	1326	1670	0.160
26	Neryl acetate	1359	1360	1699	0.330
27	α-Copaene	1374	1372	1493	3.210
28	Geranyl acetate	1379	1379	1753	0.500
29	β-Bourbonene	1387	1385	1546	0.090
30	β-Elemene	1389	1389	1591	tr
31	α-Chamipinene	1396	1397	-	tr
32	β-Caryophyllene	1408	1411	1617	2.200
33	β-Cedrene	1419	1419	1633	0.180
34	(E)-α-Ionone	1428	1426	-	0.160
35	α-Himachalene	1449	1441	1718	0.140
36	(E-β)-Farnesene	1454	1456	1668	0.080
37	Alloaromadendrene	1458	1459	1662	0.090
38	(E)-9-epi-Caryophyllene	1464	1465	-	0.080
39	Germacrene D	1484	1480	1711	0.100
40	α-Ylangene	1492	1493	1728	0.500
41	Cubebolepi	1493	1495	2037	0.050
42	*cis*-α-Bisabolene	1506	1507	1740	0.080
43	Tridecanal	1509	1508	-	tr
44	β-Curcumene	1514	1514	1756	1.590
45	δ-Cadinene	1522	1521	1785	tr
46	g-Cuprenene	1532	1527	-	tr
47	Germacrene B	1559	1560	1572	tr
48	(E)-Nerolidol	1561	1566	2044	0.100
49	Caryophyllene oxide	1582	1582	2000	0.310
50	Viridiflorol	1592	1593	2112	0.100
51	n-Hexadecane	1600	1598		tr
52	Humulene epoxide II	1608	1607	2011	0.260
53	Isolongifolan-7-α-ol	1618	1619	-	0.150
54	Aromadendrene (epoxide-allo)	1639	1638	-	0.080
55	β-Eudesmol	1649	1648	2248	tr
56	α-Cadinol	1652	1651	2224	0.070
57	α-7-epi-Eudesmol	1662	1663	2244	0.050
58	Elemol acetate	1680	1681	-	tr
59	Acorenone	1692	1694	-	tr
60	α-Bisabolol acetate	1798	1798	-	tr
61	Farnesyl acetate < 2E.6E >	1845	1844	2267	0.070
62	n-Hexadecanol	1874	1872	-	tr
63	Cedrol-diol (8S.13)	1897	1897	-	0.060
64	Methyl hexadecanoate	1921	1924	-	tr
65	Geranyl benzoate	1958	1956	-	tr
66	1-Eicosene	1987	1988	-	tr
67	n-Eicosane	2000	2000	-	tr
68	13-epi-Manool oxide	2009	2019	-	0.120
69	Isobergaptene	2033	2034	-	tr
70	13-epi-Manool	2059	2051	-	tr
71	n-Octadecanol	2077	2075	-	0.120
72	Methyl linoleate	2095	2096	-	tr
73	Abienol	2149	2151	-	0.05
74	Phenylethylcinnamate	2179	2173	-	0.060
75	n-Docosane	2200	2198	-	tr
76	Phytol acetate	2222	2218	-	0.100
77	4-epi-Abietal	2298	2298	-	tr
78	n-Pentacosane	2500	2500	-	0.280
Total identified					94.090
Monoterpene hydrocarbons (MH)					0.490
Oxygenated monoterpenes (OM)					84.980
Sesquiterpene hydrocarbons (SH)					6.180
Oxygenated sesquiterpenes (OS)					0.450
Diterpenes					0.170
Others compounds					1.820
Yield of essential oil (*w*/*w*)%					0.470

tr—trace < 0.05%; RI_ref_—theoretical retention indices of Adams book stores; RI_np_—retention index calculated on the HP-5MS nonpolar column; RI_p_—retention index calculated on the HP-Wax polar column; percentage (%)—the content of each constituent.

**Table 5:** Antioxidant activity of *M. officinalis* leaf EO.

Antioxidant Activity Methods	Essential Oil and References of Synthetic Antioxidants (n = 6)	IC_50_ (µg/mL)	β-Carotene/Linoleic Acid (%) (n = 6)
**DPPH radicals**	*M. officinalis* EO	>44,000	
	Vitamin E	9.560 ± 0.070	
	Vitamin C	7.250 ± 0.030	
	BHT	20.110 ± 0.010	
	BHA	8.350 ± 0.050	
**β-carotene/linoleic acid**	*M. officinalis* EO		15.860 ± 0.700
	BHT		83.560 ± 2.130

**Table 6:** Results of antimicrobial activity tests (diameter of inhibition zones in mm ± 1 of the essential oil of *M. officinalis* from Algeria.

Test Microorganisms	EO *M. officinalis* Leaves	Ampicillin	Nalidixic Acid	Nystatine
Gram positive Bacteria				
*Staphylococcus aureus*	-	21	-	-
*Bacillus subtilis*	10	46	-	-
Gram negative Bacteria				
*Pseudomonas aeruginosa*	50	-	29	-
*Escherichia coli*	50	-	30	-
*Klebsiella pneumonia*	10	-	18	-
*Salmonella enterica*	50	-	19	-
Yeasts				
*Candida albicans*	50	-	-	18
*Saccharomyces cerevisiae*	24	-	-	29
Filamentous fungi				
*Fusarium oxysporum albedinis*	50	-	-	20
*Fusarium oxysporum lini*	34	-	-	24
*Mucor ramannianus*	24	-	-	31

-, absence of inhibition zone detected.

## Data Availability

All data generated or analyzed are contained within the present article.

## References

[1] Abdellatif F., Boudjella H., Zitouni A., Hassani A. (2014). Chemical composition and antimicrobial activity of the essential oil from leaves of Algerian *Melissa officinalis* L.. EXCLI J..

[2] Shakeri A., Sahebkar A., Javadi B. (2016). *Melissa officinalis* L.—A review of its traditional uses, phytochemistry and pharmacology. J. Ethnopharmacol..

[3] Miraj S., Rafieian-Kopaei, Kiani S. (2017). Melissa officinalis L: A review study with an antioxidant prospective. J. Evid. Based Complement. Altern. Med..

[4] Ashori A., Hamzeh Y., Amani F. (2011). Lemon balm (*Melissa officinalis*) stalk: Chemical composition and fiber morphology. J. Polym. Environ..

[5] Moradkhani H., Sargsyan E., Bibak H., Naseri B., Sadat-Hosseini M., Fayazi-Barjin A., Meftahizade H. (2010). *Melissa officinalis* L., a valuable medicine plant: A review. J. Med. Plants Res..

[6] Carocho M., Barros L., Calhelha R.C., Ćirić A., Soković M., Santos-Buelga C., Morales P., Ferreira I.C.F.R. (2015). Melissa officinalis L. decoctions as functional beverages: A bioactive approach and chemical characterization. Food Funct..

[7] Moacǎ E.A., Farcaş C., Ghiţu A., Coricovac D., Popovici R., Cǎrǎba-Meiţǎ N.L., Ardelean F., Antal D.S., Dehelean C., Avram Ş. (2018). A comparative study of *Melissa officinalis* leaves and stems ethanolic extracts in terms of antioxidant, cytotoxic, and antiproliferative potential. Evid. Based Complement. Altern. Med..

[8] Stankovic M. (2020). Lamiaceae Species.

[9] Coelho-de-Souza A.N., Alves-Soares R., Oliveira H.D., Gomes-Vasconcelos Y.A., Souza P.J.C., Santos-Nascimento T., Oliveira K.A., Diniz L.R.L., Guimarães-Pereira J., Leal-Cardoso J.H. (2021). The essential oil of Hyptis crenata Pohl ex Benth. presents an antiedematogenic effect in mice. Braz. J. Med. Biol. Res..

[10] Dei Cas M., Ghidoni R. (2018). Cancer prevention and therapy with polyphenols: Sphingolipid-mediated mechanisms. Nutrients.

[11] Asadi A., Shidfar F., Safari M., Hosseini A.F., Fallah Huseini H., Heidari I., Rajab A. (2019). Efficacy of *Melissa officinalis* L. (lemon balm) extract on glycemic control and cardiovascular risk factors in individuals with type 2 diabetes: A randomized, double-blind, clinical trial. Phyther. Res..

[12] Hassanzadeh G., Pasbakhsh P., Akbari M., Shokri S., Ghahremani M., Amin G., Kashani I., Tameh A.A. (2011). Neuroprotective properties of *Melissa officinalis* L. extract against ecstasy-induced neurotoxicity. Cell J..

[13] Taiwo A.E., Leite F.B., Lucena G.M., Barros M., Silveira D., Silva M.V., Ferreira V.M. (2012). Anxiolytic and antidepressant-like effects of *Melissa officinalis* (lemon balm) extract in rats: Influence of administration and gender. Indian J. Pharmacol..

[14] Beloued A. (2005). Plantes Médicinales d’Algérie.

[15] Uritu C.M., Mihai C.T., Stanciu G.-D., Dodi G., Alexa-Stratulat T., Luca A., Leon-Constantin M.-M., Stefanescu R., Bild V., Melnic S. (2018). Medicinal plants of the family Lamiaceae in pain therapy: A review. Pain Res. Manag..

[16] De Sousa A.C., Gattass C.R., Alviano D.S., Alviano C.S., Blank A.F., Alves P.B. (2004). *Melissa officinalis* L. essential oil: Antitumoral and antioxidant activities. J. Pharm. Pharmacol..

[17] Mimica-Dukic N., Bozin B., Sokovic M., Simin N. (2004). Antimicrobial and antioxidant activities of *Melissa officinalis* L.(Lamiaceae) essential oil. J. Agric. Food Chem..

[18] Vanti G., Ntallis S.G., Panagiotidis C.A., Dourdouni V., Patsoura C., Bergonzi M.C., Lazari D., Bilia A.R. (2020). Glycerosome of *Melissa officinalis* L. essential oil for Effective anti-HSV type 1. Molecules.

[19] Božović M., Navarra A., Garzoli S., Pepi F., Ragno R. (2017). Esential oils extraction: A 24-hour steam distillation systematic methodology. Nat. Prod. Res..

[20] Aziz Z.A.A., Ahmad A., Setapar S.H.M., Karakucuk A., Azim M.M., Lokhat D., Rafatullah M., Ganash M., Kamal M.A., Ashraf G.M. (2018). Essential Oils: Extraction Techniques, Pharmaceutical And Therapeutic Potential—A Review. Curr. Drug Metab..

[21] Menichini F., Tundis R., Bonesi M., De Cindio B., Loizzo M.R., Conforti F., Statti G.A., Menabeni R., Bettini R., Menichini F. (2011). Chemical composition and bioactivity of *Citrus medica* L. cv. Diamante essential oil obtained by hydrodistillation, cold-pressing and supercritical carbon dioxide extraction. Nat. Prod. Res..

[22] Lee M.S., Choi J., Posadzki P., Ernst E. (2012). Aromatherapy for health care: An overview of systematic reviews. Maturitas.

[23] Sowndhararajan K., Kim S. (2016). Influence of fragrances on human psychophysiological activity: With special reference to human electroencephalographic response. Sci. Pharm..

[24] Peterfalvi A., Miko E., Nagy T., Reger B., Simon D., Miseta A., Czéh B., Szereday L. (2019). Much more than a pleasant scent: A review on essential oils supporting the immune system. Molecules.

[25] De Groot A.C., Schmidt E. (2016). Essential oils, Part I: Introduction. Dermatitis.

[26] Yavari A., Nazeri V., Sefidkon F., Hassani M.E. (2010). Influence of some environmental factors on the essential oil variability of Thymus migricus. Nat. Prod. Commun..

[27] Aboukhalid K., Al Faiz C., Douaik A., Bakha M., Kursa K., Agacka-Mołdoch M., Machon N., Tomi F., Lamiri A. (2017). Influence of environmental factors on essential oil variability in *Origanum compactum* benth. Growing wild in Morocco. Chem. Biodivers..

[28] Freeman M., Ayers C., Peterson C., Kansagara D. (2019). Aromatherapy and essential oils : A map of the evidence. VA Evidence Synthesis Program Reports.

[29] Lee M., Lee H., Khalil M., Lim H., Lim H.-J. (2018). Aromatherapy for managing pain in primary dysmenorrhea: A systematic review of randomized placebo-controlled trials. J. Clin. Med..

[30] Benarfa A., Begaa S., Messaoudi M., Hamlat N., Sawicka B. (2020). Elemental composition analysis of *Pistacia lentiscus* L., leaves collected from Mitidja plain in Algeria using instrumental neutron activation analysis (INAA) technique. Radiochim. Acta.

[31] Begaa S., Messaoudi M. (2019). Toxicological aspect of some selected medicinal plant samples collected from Djelfa, Algeria Region. Biol. Trace Elem. Res..

[32] Begaa S., Messaoudi M., Benarfa A. (2020). Statistical approach and neutron activation analysis for determining essential and toxic elements in two kinds of algerian artemisia plant. Biol. Trace Elem. Res..

[33] Ghosh A., Saleh-e-In M.M., Abukawsar M.M., Ahsan M.A., Rahim M.M., Bhuiyan M.N.H., Roy S.K., Naher S. (2019). Characterization of quality and pharmacological assessment of *Pimpinella anisum* L. (Anise) seeds cultivars. J. Food Meas. Charact..

[34] Begaa S., Messaoudi M. (2018). Thermal neutron activation analysis of some toxic and trace chemical element contents in *Mentha pulegium* L.. Radiochim. Acta.

[35] van der Ploeg R.R., Böhm W., Kirkham M.B. (1999). On the origin of the theory of mineral nutrition of plants and the law of the minimum. Soil Sci. Soc. Am. J..

[36] Hollá M., Svajdlenka E., Tekel J., Vaverková S., Havránek E. (1997). Composition of the essential oil from *Melissa officinalis* L. cultivated in Slovak Republic. J. Essent. Oil Res..

[37] Shabby A.S., El-Gengaihi S., Khattab M. (1995). Oil of *Melissa officinalis* L., as affected by storage and herb drying. J. Essent. Oil Res..

[38] Carnat A.P., Carnat A., Fraisse D., Lamaison J.L. (1998). The aromatic and polyphenolic composition of lemon balm (*Melissa officinalis* L. subsp. officinalis) tea. Pharm. Acta Helv..

[39] Aharizad S., Rahimi M.H., Moghadam M., Mohebalipour N. (2012). Study of genetic diversity in lemon balm (*Melissa officinalis* l.) populations based on morphological traits and essential oils content. Ann. Biol. Res..

[40] Adzet T., Ponz R., Wolf E., Schulte E. (1992). Content and composition of *M. officinalis* oil in relation to leaf position and harvest time. Planta Med..

[41] (1970). European Pharmacopeia. J. Am. Pharm. Assoc..

[42] Van Den Dool H., Kratz P.D. (1963). A generalization of the retention index system including linear temperature programmed gas-liquid partition chromatography. J. Chromatogr. A.

[43] Adams R.P. (2007). Identification of Essential Oil Components by Gas Chromatography/Mass Spectrometry.

[44] Braca A., Sortino C., Politi M., Morelli I., Mendez J. (2002). Antioxidant activity of flavonoids from *Licania licaniaeflora*. J. Ethnopharmacol..

[45] Shon M.Y., Kim T.H., Sung N.J. (2003). Antioxidants and free radical scavenging activity of *Phellinus baumii* (Phellinus of Hymenochaetaceae) extracts. Food Chem..

[46] Al-Saikhan M.S., Howard L.R., Miller J.C. (1995). Antioxidant activity and total phenolics in different genotypes of potato (*Solanum tuberosum*, L.). J. Food Sci..

[47] Bauer A.W. (1966). Antimicrobial susceptibility testing by a standardized single disk method. Am. J. Clin. Pathol..

[48] Hack H., Bleiholder H., Buhr L., Meier U., Schnock-Fricke U., Weber E., Witzenberger A. (1992). Einheitliche codierung der phänologischen entwicklungsstadien mono-und dikotyler pflanzen–erweiterte BBCH-Skala, Allgemein. Nachrichtenbl. Deut. Pflanzenschutzd.

[49] SAS Institute Inc. (2008). SAS/STATÒ 9.2 User’s Guide.

